# Urethane tetrathiafulvalene derivatives: synthesis, self-assembly and electrochemical properties

**DOI:** 10.3762/bjoc.11.255

**Published:** 2015-11-27

**Authors:** Xiang Sun, Guoqiao Lai, Zhifang Li, Yuwen Ma, Xiao Yuan, Yongjia Shen, Chengyun Wang

**Affiliations:** 1 Key Laboratory for Advanced Materials and Institute of Fine Chemicals, East China University of Science and Technology, 130 Meilong Road, Shanghai 200237, China; 2 Key Laboratory of Organosilicon Chemistry and Material Technology of Ministry of Education, Hangzhou Normal University, Hangzhou 310012, China

**Keywords:** hydrogen bond, nanoribbon, self-assembly, tetrathiafulvalene, urethane

## Abstract

This paper reports the self-assembly of two new tetrathiafulvalene (TTF) derivatives that contain one or two urethane groups. The formation of nanoribbons was evidenced by scanning electron microscopy (SEM) and X-ray diffraction (XRD), which showed that the self-assembly ability of **T_1_** was better than that of **T_2_**. The results revealed that more urethane groups in a molecule did not necessarily instigate self-assembly. UV–vis and FTIR spectra were measured to explore noncovalent interactions. The driving forces for self-assembly of TTF derivatives were mainly hydrogen bond interactions and π–π stacking interactions. The electronic conductivity of the **T_1_** and **T_2_** films was tested by a four-probe method.

## Introduction

In recent years, there has been an enormous increase of interest in functional organic nanomaterials, given that they are promising materials with a variety of applications including optoelectronic and bioelectronic devices [1–2]. The mechanism behind the formation of functional organic nanomaterials is generally accepted to be the self-assembly of supermolecules, which is constructed through weak noncovalent interactions such as π–π stacking, van der Waals interactions, charge transfer and H-bonding interactions [3–6]. Generally speaking, H-bonding interactions are the key intermolecular interactions in molecular self-assembly systems. Therefore, molecules containing urea, amide and other similar groups have been investigated because these molecules can easily generate intermolecular hydrogen bonds [7–9].

Tetrathiafulvalene (TTF) derivatives have been widely investigated in the fields of supramolecular and materials chemistry due to their great potential application in molecular electronics, for example, as switches and conductors [10–14]. As we all know, TTF derivatives can form charge transfer (CT) complexes with electron acceptors such as tetracyanoquinodimethane (TCNQ), and the CT complexes of TTF derivatives and TCNQ exhibit high electrical conductivity [14–16]. Therefore, TTF derivatives are extensively used in the field of functional organic conductive nanomaterials.

**Figure 1:**
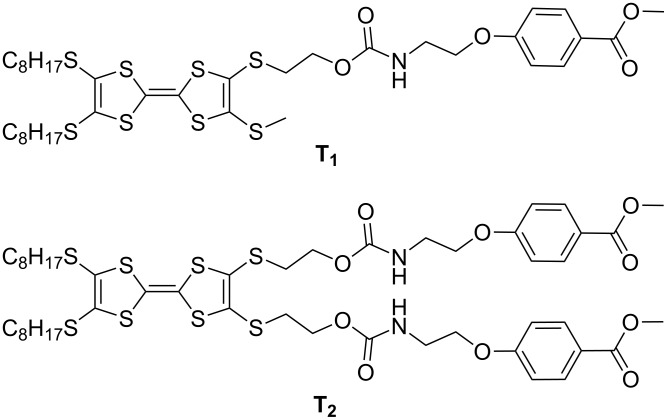
Molecular structure of TTF derivatives **T_1_** and **T_2_**.

Herein, we designed and synthesized two compounds, **T_1_** and **T_2_**, which contain TTF units and urethane groups (Figure 1). The combination of the urethane group (forming hydrogen bonds) and the TTF unit (forming π–π stacking) may promote the formation of nanostructures. To the best of our knowledge, urethane groups have been rarely introduced into the molecular structure of TTF derivatives to generate an H-bonding chain.

## Results and Discussion

### Synthesis and characterization

**Scheme 1:**
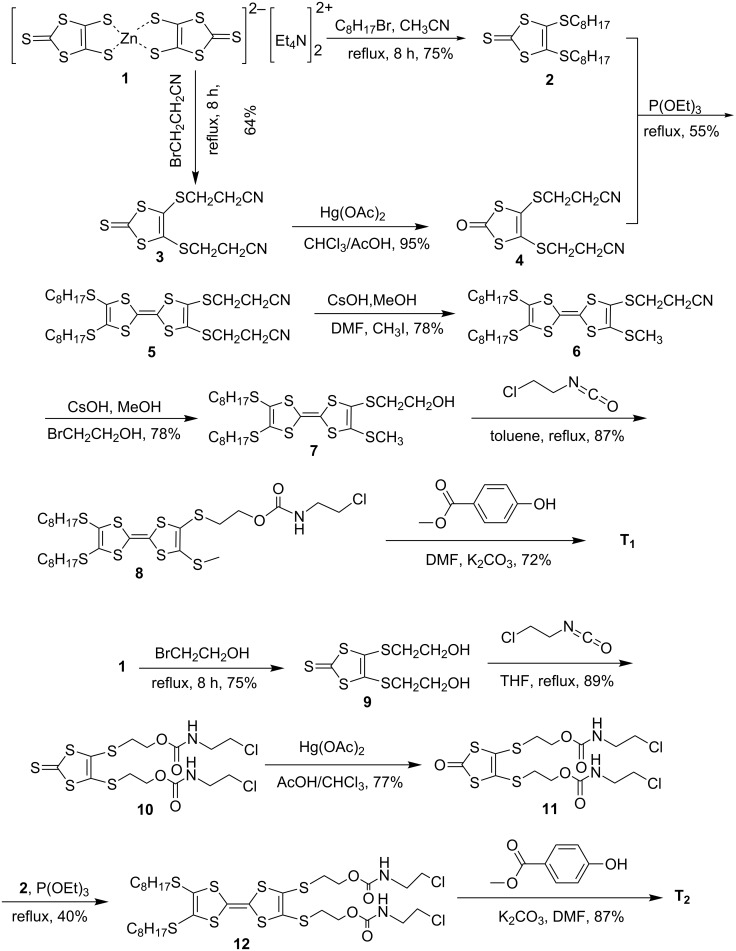
The synthetic routes of compounds **T_1_** and **T_2_**.

The synthetic routes for two newly designed TTF derivatives containing one or two urethane groups are shown in Scheme 1. Compounds **2** [17], **3** [18], **4** [19], **5** [19], **6** [18,20] and **7** [18,21] were synthesized from commercially available starting materials according to the reported methods. Compound **8** [18,21] was obtained by the reaction of **7** with 2-chloroethyl isocyanate in dry and degassed toluene. Finally, the TTF derivative **T_1_** was obtained in acceptable yield (72%). For the synthesis of **T_2_**, urethane groups were introduced first, and then the coupling reaction was carried out. The new compounds **T_1_** and **T_2_** were characterized by ^1^H, ^13^C NMR, HRMS–ESI (for the spectra see Supporting Information File 1) and elemental analysis. In addition, other intermediates previously reported in the literature were also characterized by ^1^H NMR, ^13^C NMR, and EIMS.

### Self-assembly and SEM investigation of **T_1_** and **T_2_**

**Figure 2:**
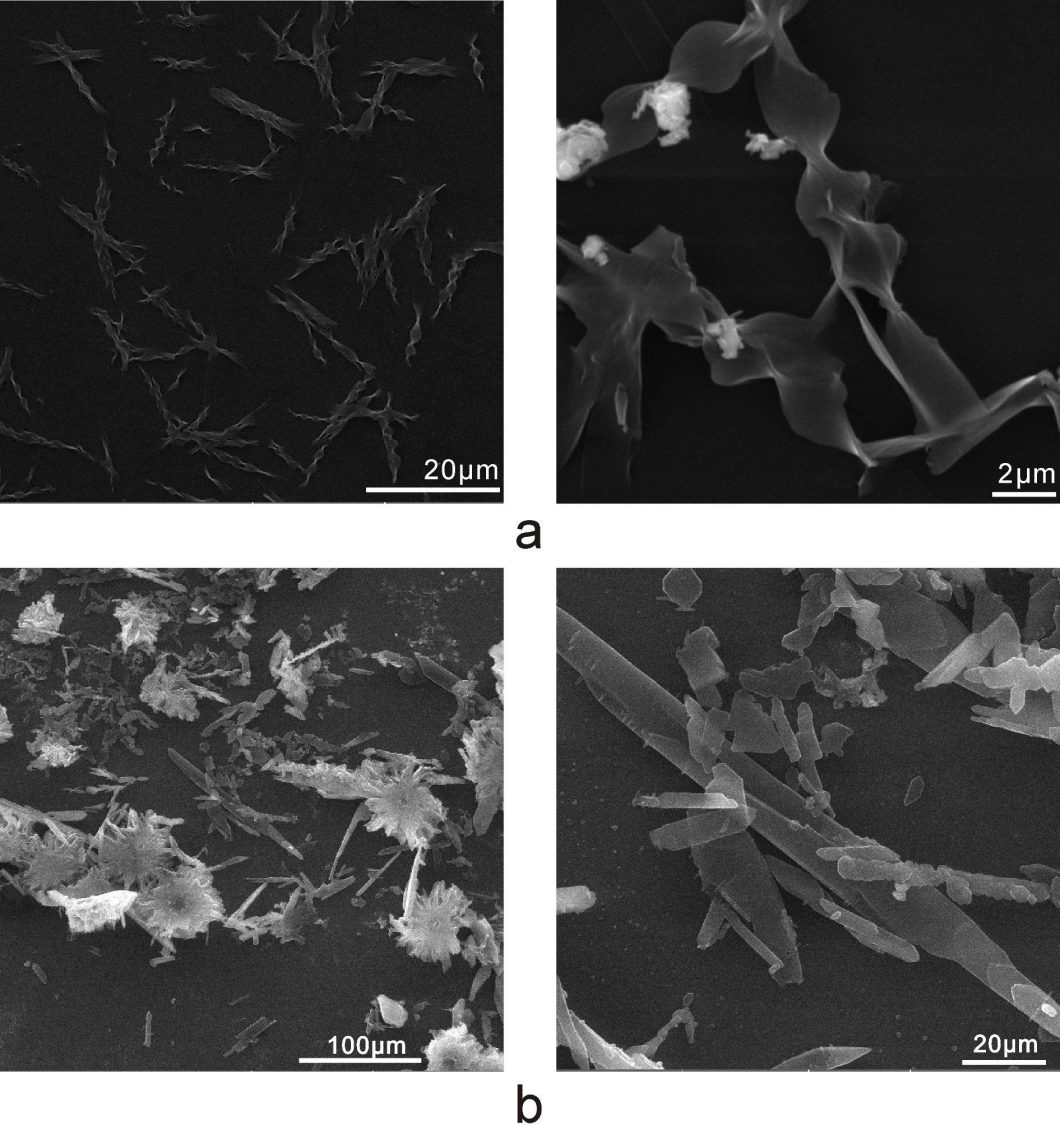
SEM images of **T_1_** (a) and **T_2_** (b) films on glass substrates (drop-coated from diluted **T_1_** or **T_2_** solution).

The studies showed that **T_1_** and **T_2_** gels were not formed in several common solvents such as hexane, chloroform, dichloromethane, tetrahydrofuran, toluene, diethyl ether, acetone, dimethylformamide, ethanol, methanol and acetonitrile when they were heated and cooled by the methods reported in the literature [2–4]. A loose gel of **T_1_** was observed in ethyl acetate when the concentration was increased to 20 mg/mL. However, the precipitate of **T_2_** was obtained under the same conditions. Moreover, their micromorphology was recorded with SEM images (Figure 2). The samples were prepared by different methods (drop-coating, spin-coating). The experiments were performed as follows: the solid compounds were completely dissolved in ethyl acetate while heating, then cooled to room temperature. The studies showed that drop-coating was better than direct spin-coating, likely because slow solvent evaporation is more conducive to the formation of regular structure. The SEM images of the **T_1_** films (Figure 2a, drop-coated from a diluted **T_1_** solution) showed that regular helical nanoribbons were observed. The diameter of the nanoribbons was approximately 500 nm with a length of >20 μm. Although nanoribbons were observed in the SEM images of **T_2_** (Figure 2b), they showed no similar ordered structure.

In addition, the X-ray diffraction (XRD) patterns of **T_1_** and **T_2_** nanoribbons were taken (Supporting Information File 1, Figure S7). The XRD pattern of **T_1_** showed three sharp peaks at 7.4°, 14.9° and 22.1°, which suggested that a lamellar stacking organization was formed [4]. This was not the case for the XRD pattern of **T_2_**. In general, intermolecular hydrogen bonding is the main driving force behind self-assembly. Although **T_2_** contains two urethane groups and **T_1_** contains one urethane group, the self-assembly ability of **T_2_** is not better than that of **T_1_**. We concluded that more intramolecular hydrogen bonds were formed in molecules of **T_2_** instead of intermolecular hydrogen bonds in ethyl acetate, which was not conducive to form regular nanoribbons.

### UV–vis and FTIR spectroscopy

**Figure 3:**
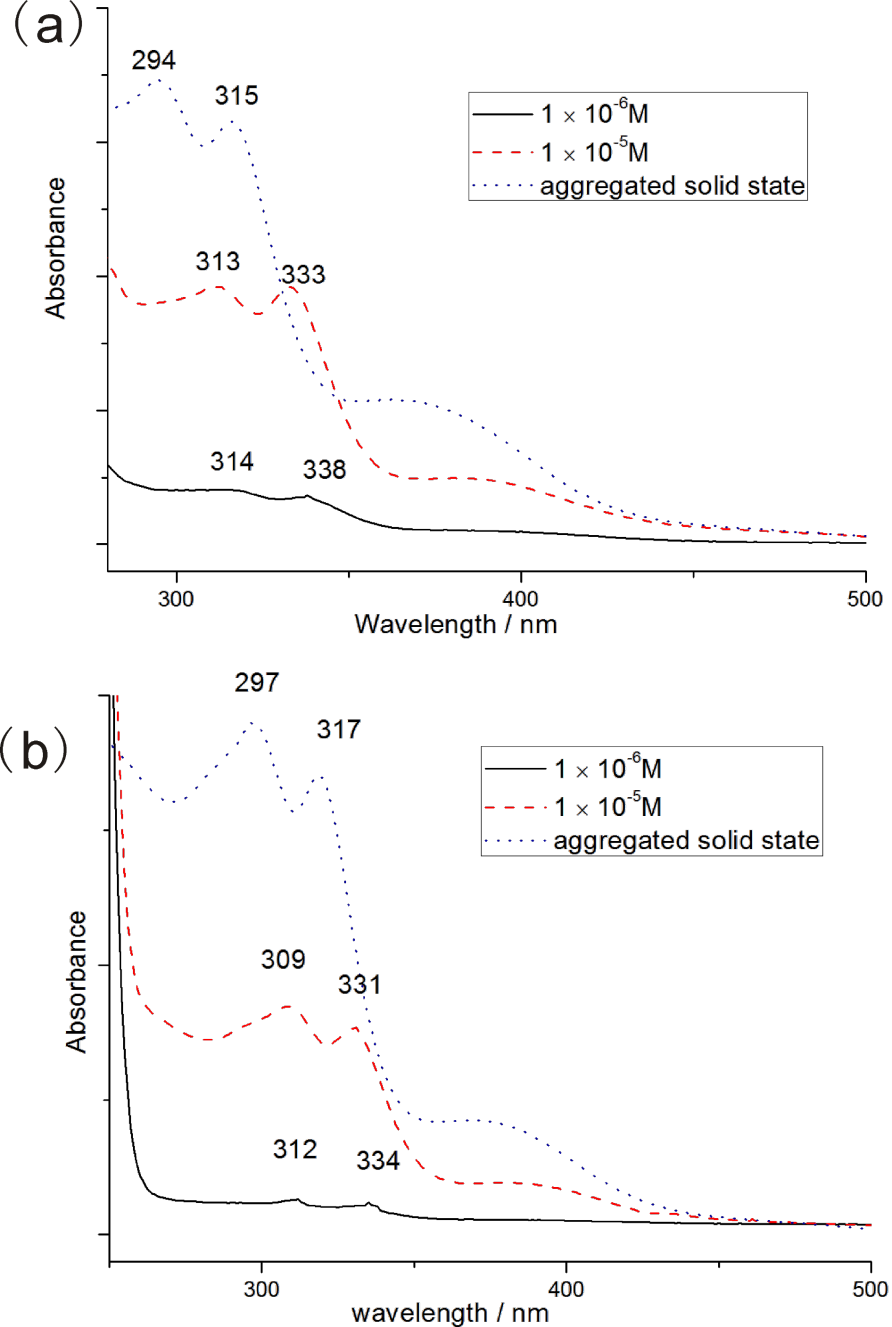
The UV–vis spectra of **T_1_** (a) and **T_2_** (b) at different concentrations in ethyl acetate.

**Figure 4:**
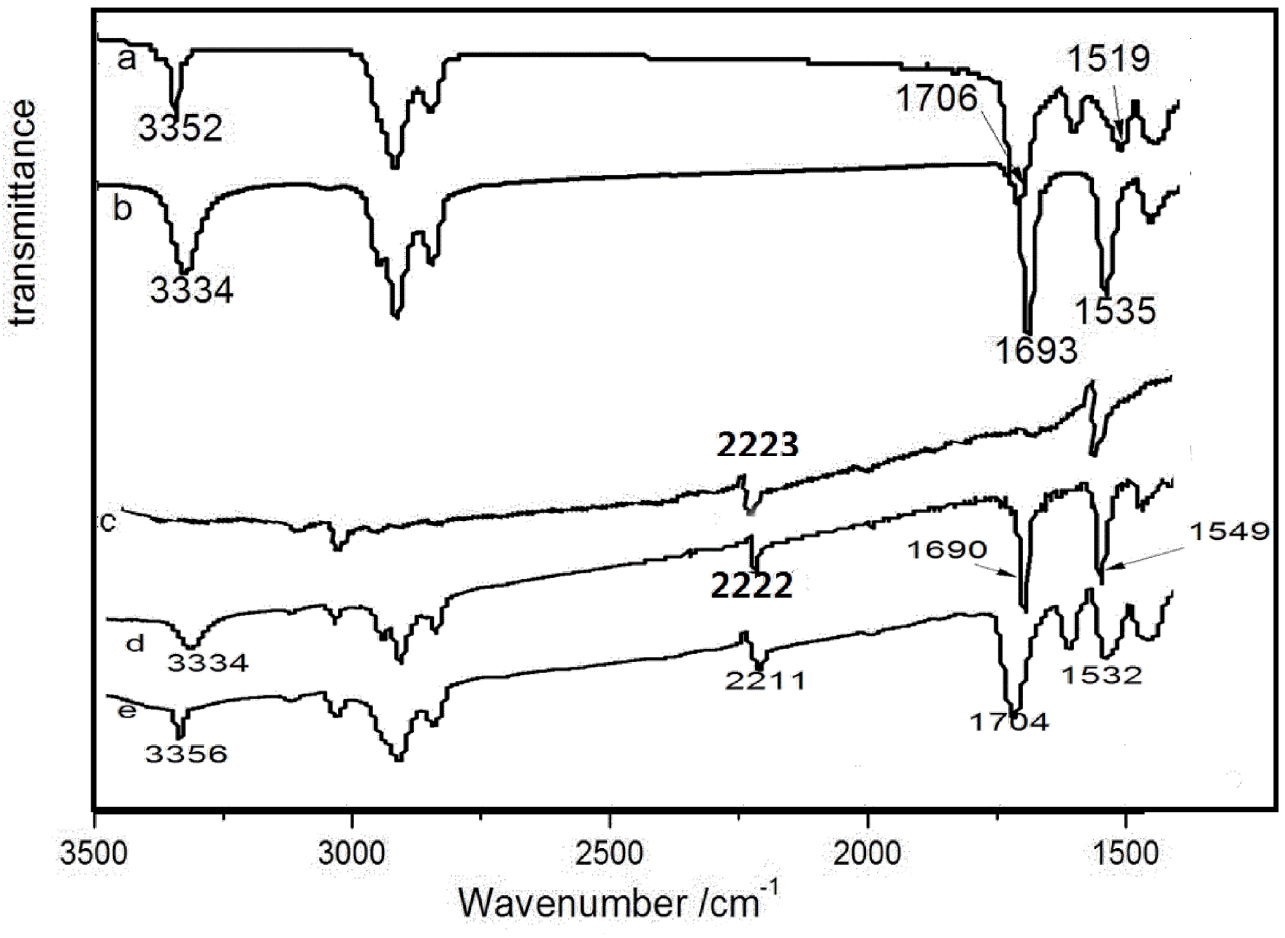
IR spectra of (a) **T_1_**, (b) **T_2_**_,_ (c) TCNQ, (d) **T_2_**/TCNQ, and (e) **T_1_**/TCNQ.

To study the intermolecular interactions, the UV–vis absorption spectra of **T_1_** and **T_2_** in ethyl acetate at different concentrations were measured (Figure 3a,b). Figure 3a shows that the two absorption peaks of **T_1_** are blue-shifted from 314 nm and 338 nm (1 × 10^−6^ M) to 294 nm and 315 nm (aggregated solid state). This was also observed for **T_2_**, which illustrated that π–π interactions and H-aggregation occurred with the increase in concentration [22–24]. To further study the driving forces for the self-assembly of **T_1_** and **T_2_**, FTIR spectra were also measured (Figure 4a,b). The FTIR spectra of **T_1_** showed an absorption peak at 3352 cm^−1^ for the N–H stretching vibration, 1706 cm^−1^ for amide I and 1519 cm^−1^ for amide II related to the urethane groups. The same situation was observed for **T_2_**. The absence of a free N–H stretching vibration (around 3400 cm^−1^) and a free C=O stretching vibration (around 1720 cm^−1^) suggested that strong hydrogen bonds between urethane groups were formed [25–26]. These results indicated that π–π interactions and hydrogen bonding were the main driving forces behind the self-assembly.

**Figure 5:**
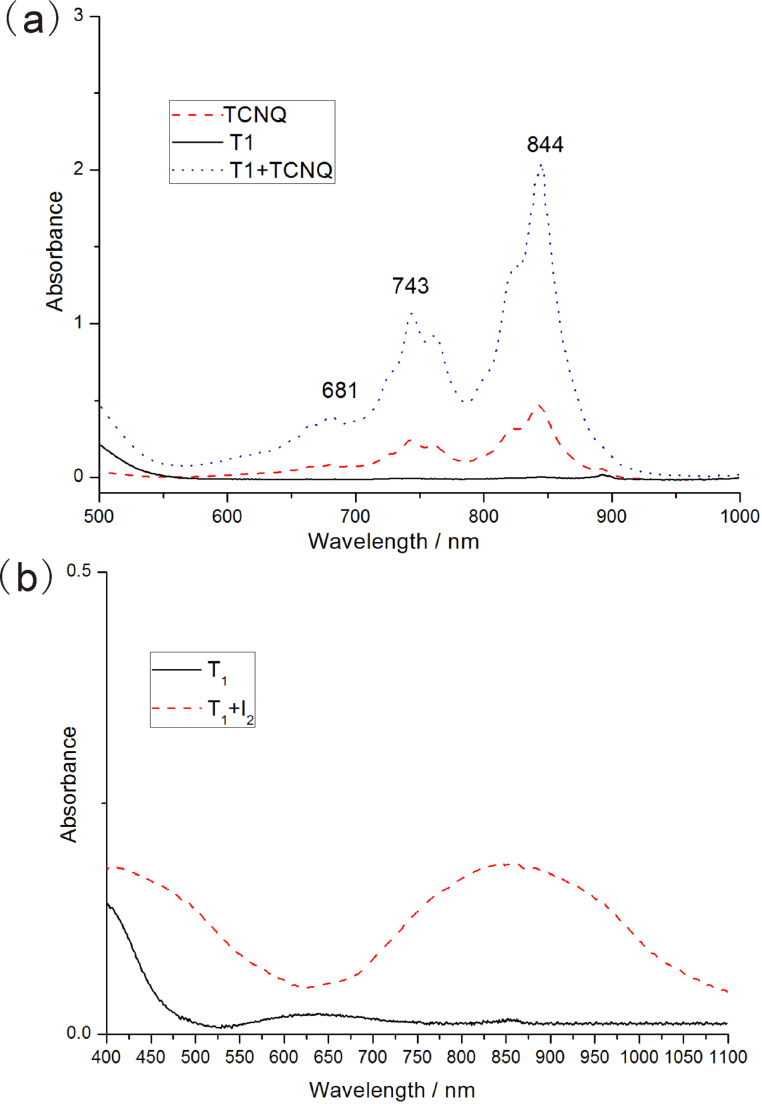
(a) UV–vis spectra of **T_1_** solutions TCNQ and **T_1_**/TCNQ in ethyl acetate (1 × 10^−3^ M). (b) UV–vis spectra of **T_1_** before and after iodine doping for 30 min.

In addition, UV−vis and FTIR spectra were measured to explore the formation of the charge-transfer complexes. TTF derivates are representative electron donors, while TCNQ is a typical electron acceptor. When one equivalent of TCNQ was added to the solution of **T_1_** in ethyl acetate, TCNQ radical anion species (TCNQ^•−^) and TTF radical cation species (TTF^•+^) were formed, which was possibly supported by the increase of the absorption bands around 600–900 nm (Figure 5a) [2,4]. Moreover, the UV–vis spectra of self-assembled nanoribbons doped with iodine were collected. It was concluded that the assembled solid structures were maintained. Figure 5b shows the UV–vis spectrum of **T_1_** (thin film on glass) before and after iodine doping. Upon exposure to iodine vapor for 30 min in a sealed container, a new absorption band was observed at approximately 850 nm, which suggested the formation of the CT complex [27].

IR spectra of TCNQ, **T_1_**/TCNQ, and **T_2_**/TCNQ are shown in Figure 4c–e. In contrast to those of **T_1_** and **T_2_**, the N–H and C=O stretching bands of the amide groups were not obviously shifted after doping with TCNQ. This indicated that the doping did not change the hydrogen-bonded structures.

### Cyclic voltammetry (CV)

**Figure 6:**
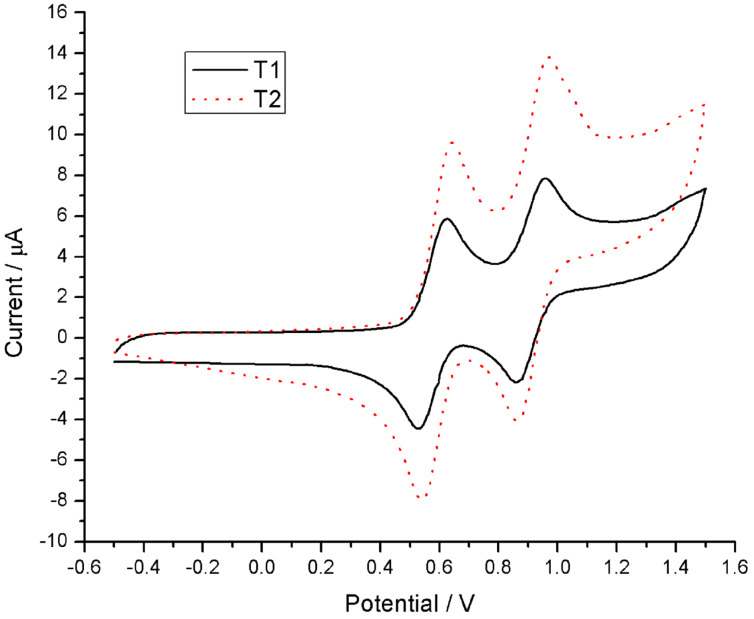
Cyclic voltammograms of **T_1_** and **T_2_** in DCM. Conditions: 0.1 M tetrabutylammonium hexafluorophosphate, 100 mV s^−1^, Ag/AgCl as the reference electrode, Pt wire as the counter electrode, and glassy carbon as the working electrode; measured under argon at 20 °C. Concentration: 1 mM for **T_1_** and 1 mM for **T_2_**.

The cyclic voltammetry experiments were carried out to explore the electrochemical properties of the TTF compounds. The cyclic voltammograms of **T_1_** and **T_2_** were measured in dry and degassed dichloromethane solution [28]. Both **T_1_** and **T_2_** displayed two, reversible, one-electron redox couples, in which the first oxidation at 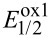 = +0.628 V (**T_1_**) and +0.643 V (**T_2_**) (vs Ag/AgCl) was in the anodic window. This indicated the successive reversible oxidation of neutral TTF (TTF^0^) to the radical cation (TTF^•+^). The second oxidation at 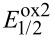 = +0.958 V (**T_1_**) and +0.973 V (**T_2_**) (vs Ag/AgCl) corresponded to the reversible oxidation of the radical cation (TTF^•+^) to the dication (TTF^2+^) (Figure 6). Both the first-wave and the second-wave oxidation potentials of **T_2_** were higher (15 mV) than those of **T_1_**, which indicated that introduction of another urethane group resulted in a decrease of the electron-donating ability.

**Figure 7:**
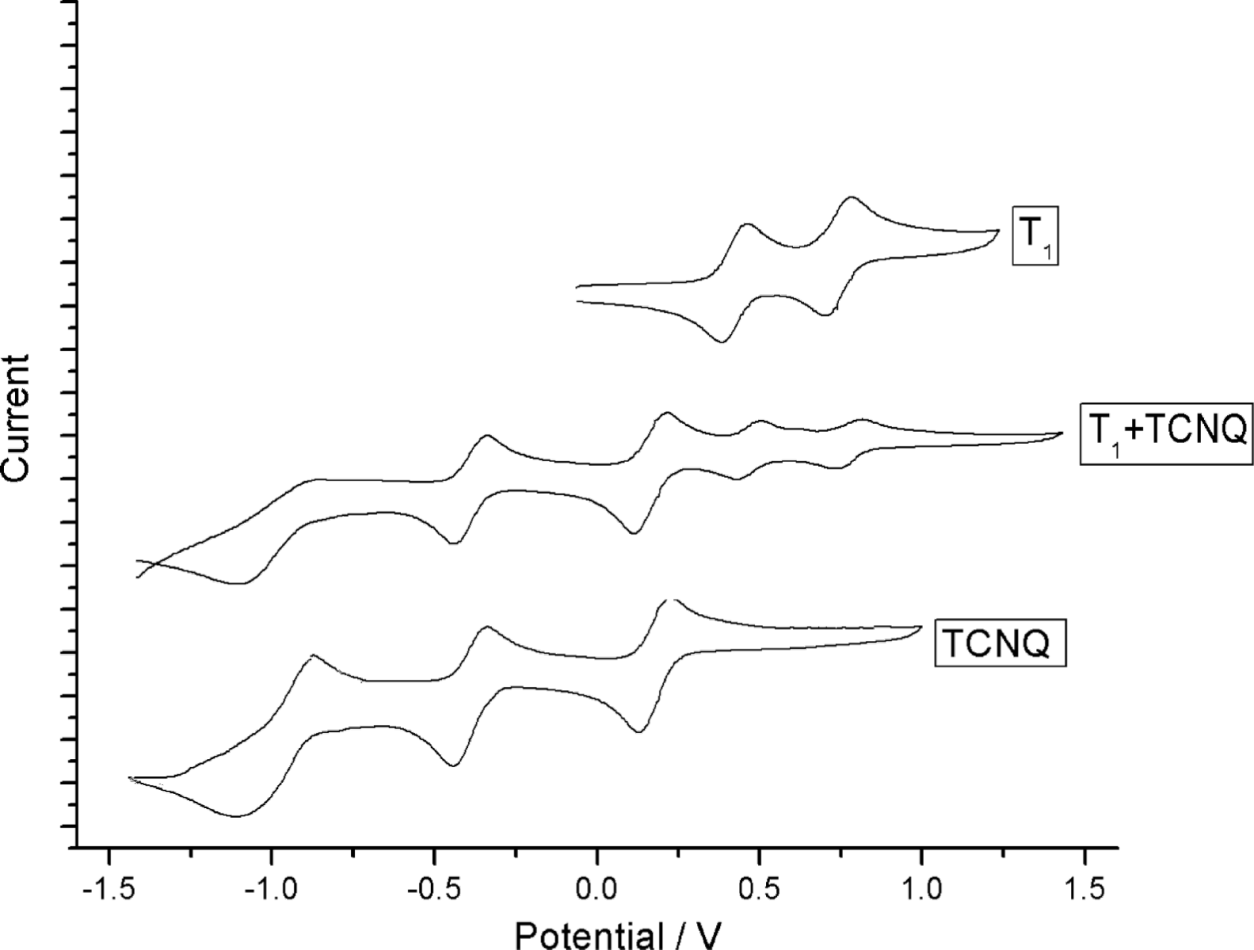
Cyclic voltammograms of **T_1_** and TCNQ in DCM. Conditions: 0.1 M tetrabutylammonium hexafluorophosphate, 100 mV s^−1^, saturated calomel electrode (SCE) as the reference electrode, Pt wire as the counter electrode, and glassy carbon as the working electrode; measured under argon at 20 °C. Concentration: 1 mM for **T_1_** and 1 mM for TCNQ.

Cyclic voltammograms were also measured to explore the formation of the charge-transfer complex. For the mixture of **T_1_** and TCNQ, five oxidation potentials at 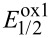 = −0.956 V (I), 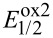 = −0.368 V (II), 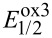 = +0.221 V (III), 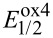 = +0.527 V (IV), and 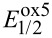 = +0.852 V (V) (vs saturated calomel electrode, SCE) were clearly discernible (Figure 7). The first three oxidation potentials belonged to TCNQ^2−^/TCNQ^−^ (I), TCNQ^−^/TCNQ^0^ (II) and TCNQ^0^/TCNQ^+^ (III), which were all lower than those of TCNQ (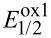 = −0.954 V(I), 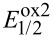 = −0.341 V(II), 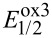 = +0.224 V(III)). The (IV) and (V) processes could be assigned to TTF^•+^/TTF^0^ (IV) and TTF^•2+^/ TTF^•+^ (V), which were all higher than those of **T_1_** (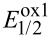 = +0.514 V (I), 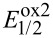 = +0.841 V (II)). These changes indicated the formation of the CT complex.

### Electrical conductivity measurements

The electrical conductivity of thin films obtained from the **T_1_** and **T_2_** samples with TCNQ (1:1 molar)/I_2_ (30 min) were further evaluated. To eliminate the influence of contact resistance, the four-probe method was carried out instead of the two-probe method [29–30]. To prepare the thin films, a diluted ethyl acetate solution was dropcasted onto a glass substrates (20 mm × 20 mm) and dried overnight at 40 °C under vacuum. The **T_1_** and **T_2_** films in the neutral state before doping behaved as typical, undoped semiconductors (σ < 10^−9^ S cm^−1^) at room temperature. Nevertheless, for **T_1_**, the conductivity increased to 5.8 × 10^−6^ S cm^−1^ when doped with TCNQ and to 3.0 × 10^−6^ S cm^−1^ when exposed to iodine vapor. As for **T_2_**, the results were 6.3 × 10^−7^ S cm^−1^ when doped with TCNQ and 1.8 × 10^−7^ S cm^−1^ when exposed to iodine vapor. These results indicated their CT complexes can function as semiconducting materials.

## Conclusion

In summary, we demonstrated that **T_2_** (containing two urethane groups) formed amorphous structures while **T_1_** (possessing one urethane group) formed nanoribbons. The self-assembly ability of **T_1_** was better than that of **T_2_**, and the results revealed that more urethane groups in a molecule did not necessarily lead to more efficient self-assembly. This may be associated with the formation of intramolecular hydrogen bonds in the **T_2_** molecule. The formation of hydrogen bonds between urethane groups and the π–π stacking interaction from TTF units were regarded as the main driving forces behind the self-assembly process. Cyclic voltammetry showed that the TTF derivatives underwent two reversible oxidation processes. In addition, the doping of nanoribbons by TCNQ/iodine resulted in the formation of charge transfer states exhibiting semiconducting properties. There is significant potential for the application of the conducting nanoribbons in molecular electronics devices.

## Supporting Information

File 1 Experimental section and copies of ^1^H, ^13^C NMR spectra, MS and XRD pattern of **T_1_** and **T_2_**.
