# Supplementary material for: Urethane tetrathiafulvalene derivatives: synthesis, self-assembly and electrochemical properties
Source: Beilstein J Org Chem. 2015 Nov 27;11:2343–9. doi: 10.3762/bjoc.11.255 (PMC4685907; doi:10.3762/bjoc.11.255)
Supplement: File 1 — Experimental section and copies of 1H, 13C NMR spectra, MS and XRD pattern of T1 and T2. [file Beilstein_J_Org_Chem-11-2343-s001.pdf]

Supporting Information

for

Urethane tetrathiafulvalene derivatives: synthesis, self-assembly and electrochemical properties

Xiang Sun[1], Guoqiao Lai[2], Zhifang Li[2], Yuwen Ma[1], Xiao Yuan[1], Yongjia Shen[1] and Chengyun Wang*[1]

Address: [1]Key Laboratory for Advanced Materials and Institute of Fine Chemicals, East China University of Science and Technology, 130 Meilong Road, Shanghai 200237, China and [2]Key Laboratory of Organosilicon Chemistry and Material Technology of Ministry of Education, Hangzhou Normal University, Hangzhou 310012, China

E-mail: Chengyun Wang - cywang@ecust.edu.cn

* Corresponding author

Experimental section and copies of ^{1}H, ^{13}C NMR spectra, MS and XRD pattern of T_1 and T_2

Contents:

1. Experimental section S3-S12
2. **Figure S1:** The ^{1}H NMR spectra of $\mathbf{T_1}$ in $CDCl_3$. S13
3. **Figure S2:** The ^{13}C NMR spectra of $\mathbf{T_1}$ in $CDCl_3$. S13
4. **Figure S3:** The HRMS of $\mathbf{T_1}$. S14
5. **Figure S4:** The ^{1}H NMR spectra of $\mathbf{T_2}$ in $CDCl_3$. S14
6. **Figure S5:** The ^{13}C NMR spectra of $\mathbf{T_2}$ in $CDCl_3$. S15
7. **Figure S6:** The HRMS of $\mathbf{T_2}$. S15
8. **Figure S7** XRD diffraction patterns of $\mathbf{T_1}$ and $\mathbf{T_2}$. S16

Experimental section

Materials and measurements

Unless otherwise stated, all commercial solvents and reagents were used as supplied without further purification. Solvents for chemical synthesis such as tetrahydrofuran (THF), dichloromethane (DCM), ethyl acetate (EA) and toluene were purified by dehydration and distilled with standard methods. The nuclear magnetic resonance (NMR) spectra were measured using a Bruker Avance III 400 spectrometer (in $CDCl_3$ and DMSO-d_6). Mass spectra were obtained using Micromass LCTTM (HRESI-TOF) spectrometer. FTIR spectra were obtained by Nicolet 380 in KBr pellets. UV–vis spectra were measured using a Nicolet CARY 100 UV–vis spectrometer (EA as solvent). SEM images were obtained by VEGA 3 TESCAN. Cyclic voltammetry was performed with a VERSA STAT II instrument (DCM as solvent). Electrical conductivity measurement was performed with SX1934 (sz-82). Elemental analyses were measured by using a VARIO EL III instrument. X-ray diffraction (XRD) analysis was performed using Rigaku D/max 2550 VB/PC apparatus.

Synthetic procedures and characterizations

Zincate (1): Compound **1** was obtained in a similar manner as described in [1]. m. p. 202-204 ^oC. ^{13}C NMR (100 MHz, $CDCl_3$, 25 ^oC) δ ppm: 209.4, 136.2, 53.1, 7.7.

4,5-Bis(octylthio)-1,3-dithiole-2-thione (2): Compound **2** was synthesized in a similar manner as described in [2]. A mixture of compound **1** (6.011 g, 8.7 mmol) and bromooctane (11.6 mL, 67.0 mmol) was dissolved in acetonitrile (120 mL). The resulting bright red solution was heated to reflux for 8 h. After

cooling to room temperature, the mixture was filtered and the solid was washed with dichloromethane. The combined organic phase was concentrated and the residue was purified by column chromatography (petroleum ether/dichloromethane 5:1) to give compound **2** as a yellow solid (2.612 g, 75%). m.p. 52-54 ^{o}C; ^{1}H NMR [400 MHz, $CDCl_3$, 25 ^{o}C] δ ppm: 2.89 (t, J = 8.0 Hz, 4H, -S-CH_2-), 1.68 (m, 4H, alkyl-H), 1.45-1.40 (m, 4H, alkyl), 1.30-1.25 (m, 16H, alkyl-H), 0.91 (t, J = 6.0 Hz, 6H,-CH_3); ^{13}C NMR (100 MHz, $CDCl_3$, 25 ^{o}C): 211.5 (-C=S), 136.4 (-C=C-), 31.8 (alkyl-C), 29.2 (alkyl-C), 29.1 (alkyl-C), 28.6 (alkyl-C), 22.7 (alkyl-C), 14.1 (alkyl-C). MS (EI, m/z) = 422.2 (M^+).

3,3'-((2-Thioxo-1,3-dithiole-4,5-diyl)bis(sulfanediyl))dipropanenitrile (3): Compound **3** was obtained in a similar manner as described in [3]. A mixture of compound **1** (4.621 g, 6.4 mmol) and bromopropionitrile (3.2 mL, 38.7 mmol) was dissolved in acetonitrile 75 mL). The resulting bright red solution was heated under reflux for 8 h. After cooling to room temperature, the mixture was filtered and the solid was washed with ethyl acetate. The combined organic phase was concentrated and the residue was purified by column chromatography (petroleum ether/ethyl acetate 1:1) to give compound **3** as a yellow solid (1.210 g, 64%). m.p. 84-85 ^{o}C; ^{1}H NMR [400 MHz, $CDCl_3$, 25 ^{o}C] δ ppm: 3.17 (t, J = 8.0 Hz, 4H, -CH_2CN), 2.83 (t, J = 8.0 Hz, 4H, -SCH_2-); ^{13}C NMR (100 MHz, $CDCl_3$, 25 ^{o}C): 211.9 (-C=S), 137.8 (-C=C-), 117.4 (-CN), 31.8 (alkyl-C), 29.5 (alkyl-C). MS (EI, m/z) = 304.1 (M^+).

3,3'-((2-Oxo-1,3-dithiole-4,5-diyl)bis(sulfanediyl))dipropanenitrile (4): Compound **4** was synthesized in a similar manner as described in [4]. Mercury acetate (3.511 g, 11 mmol) was added to a solution of compound **3**

(1.235 g, 4 mmol) in chloroform-acetate acid (3:1, 60 mL) at room temperature and the mixture was stirred overnight. The mixture was filtered and the solid was washed with dichloromethane. The combined organic phase was washed with water (2 × 30 mL), saturated sodium bicarbonate solution (3 × 30 mL), and brine (50 mL), and then dried over anhydrous sodium sulfate. The organic solvent was removed with a rotavapor to give compound **4** as a faint yellow solid (1.105 g, 95%). m.p. 86-87 ^oC; ^1H NMR [400 MHz, $CDCl_3$, 25 ^oC] δ ppm: 3.16 (t, J = 8.0 Hz, 4H, $-CH_2CN$), 2.83 (t, J = 8.0 Hz, 4H, $-SCH_2-$); ^{13}C NMR (100 MHz, $CDCl_3$, 25 ^oC): 189.9 (-C=O), 137.8 (-C=C-), 117.4 (-CN), 31.8 (alkyl-C), 29.5 (alkyl-C). MS (EI, m/z): = 288.2 (M^+).

3,3'-((4',5'-Bis(octylthio)-[2,2'-bi(1,3-dithiolylidene)]-4,5-diyl)bis(sulfane-diyl))dipropanenitrile (5): Compound **5** was obtained in a similar manner as described in [4]. The mixture of compound **2** (4.101 g, 10 mmol) and compound **4** (2.921 g, 10 mmol) was dissolved in triethyl phosphite (10 mL) and then the mixture was heated at 110 ^oC under nitrogen for 12 h. After cooling to room temperature, the solvent was removed with a rotavapor and the resulting residual was purified by column chromatography (eluent: dichloromethane) to give compound **5** as a bright red solid (3.714 g, 55%). m.p. 108-110 ^oC; ^1H NMR [400 MHz, $CDCl_3$, 25 ^oC] δ ppm: 3.09 (t, J = 8.0 Hz, 4H, $-CH_2CN$), 2.83 (t, J = 6.0 Hz, 4H, $-SCH_2$), 2.75 (t, J = 8.0 Hz, 4H, $-SCH_2-$), 1.68 (m, 4H, alkyl-H), 1.45-1.40 (m, 4H, alkyl-H), 1.33-1.25 (m, 16H, alkyl-H), 0.88 (t, J = 6.0 Hz, 6H, $-CH_3$). ^{13}C NMR (100 MHz, $CDCl_3$, 25 ^oC): 127.9 (-C=C-), 127.8 (-C=C-), 117.4 (-CN), 114.1(-C=C-), 106.1(-C=C-), 31.8 (alkyl-C), 29.2 (alkyl-C), 29.1 (alkyl-C), 28.6 (alkyl-C), 22.7 (alkyl-C), 14.1 (alkyl-C). MS (EI, m/z) = 662.1 (M^+).

3-((5-(Methylthio)-4',5'-bis(octylthio)-[2,2'-bi(1,3-dithioly-lide-ne)]-4-yl)thi-o)propanenitrile (6): Compound **6** was obtained in a similar manner as described in [3,5]. Cesium hydroxide monohydrate (0.036 g, 0.21 mmol) in dry methanol (10 mL) was added to compound **5** (0.131 g, 0.198 mmol) dissolved in dry and degassed DMF (50 mL). The reaction mixture was stirred during 10 min, the color becoming dark red. Then, an excess of iodomethane (0.100 g) was added in one portion. The color of the reaction mixture turned back to orange, and the reaction mixture was stirred at room temperature for 2 h. The solvent was removed in vacuum, and then the residue was dissolved in dichloromethane (50 mL), washed three times with water and dried over anhydrous sodium sulfate. The mixture was concentrated in vacuum and the residue was purified by chromatography on a silica gel column (eluent: petroleum ether/dichloromethane 2:1). Compound **6** was obtained in 78% yield (0.096 g) as a bright red solid. m.p. 90-92 ^oC; ^1H NMR [400 MHz, $CDCl_3$, 25 ^oC] δ ppm: 3.03 (t, J = 8.0 Hz, 2H, $-CH_2CN$), 2.82 (t, J = 8.0 Hz, 4H,$-SCH_2-$), 2.71 (t, J = 8.0 Hz, 2H, $-SCH_2-$), 2.47 (s, 3H, $-CH_3$), 1.61 (m, 4H, alkyl-H), 1.33-1.25 (m, 20H, alkyl-H), 0.88 (t, J = 6.0Hz, 6H,$-CH_3$). ^{13}C NMR (100 MHz, $CDCl_3$, 25 ^oC): 127.9 (-C=C-), 127.8 (-C=C-), 117.4 (-CN), 114.1(-C=C-), 106.1(-C=C-), 31.8 (alkyl-C), 29.2 (alkyl-C), 29.1 (alkyl-C), 28.6 (alkyl-C), 22.7 (alkyl-C), 14.1 (alkyl-C). MS (EI, m/z) = 623.1 (M^+).

2-((5-(Methylthio)-4',5'-bis(octylthio)-[2,2'-bi(1,3-dithiolylidene)]-4-yl)thio)ethanol (7): Compound **7** was synthesized in a similar manner as described in [3,6]. Cesium hydroxide monohydrate (0.190 g, 1.1 mmol) in dry methanol (10 mL) was added to compound **6** (0.630 g, 1 mmol) dissolved in dry and degassed DMF (50 mL). The reaction mixture was stirred during 10

min, the color becoming dark red. Then, an excess of bromoethanol (2 mL) was added in one portion. The color of the reaction mixture turned back to orange, and the reaction mixture was stirred at room temperature for 12 h. The solvent was removed in vacuum, and then the residue was dissolved in dichloromethane (50 mL), washed three times with water and dried over anhydrous sodium sulfate. The mixture was concentrated in vacuum and the residue was purified by chromatography on a silica gel column (eluent: petroleum ether/dichloromethane 2:1). Compound **7** was obtained in 78% yield (0.480 g) as a bright red solid. m.p. 89-91 ^oC; ^1H NMR [400 MHz, $CDCl_3$, 25 ^oC] δ ppm: 3.74 (t, J = 7.0 Hz, 2H, $-CH_2OH$), 2.95 (t, J = 7.0 Hz, 2H,$-SCH_2-$), 2.82 (t, J = 7.0 Hz, 4H, $-SCH_2-$), 2.48 (s, 3H, $-CH_3$), 1.63 (m, 4H, alkyl-H), 1.41-1.25 (m, 20H, alkyl-H), 0.89 (t, J = 6.0 Hz, 6H, $-CH_3$). ^{13}C NMR (100 MHz, $CDCl_3$, 25 ^oC): 127.9 (-C=C-), 127.8 (-C=C-), 114.1(-C=C-), 106.1(-C=C-), 65.1($-CH_2-OH$), 31.8 (alkyl-C), 29.2 (alkyl-C), 29.1 (alkyl-C), 28.6 (alkyl-C), 22.7 (alkyl-C), 14.1 (alkyl-C). MS (EI, m/z) = 614.1 (M^+).

2-((5-(Methylthio)-4',5'-bis(octylthio)-[2,2'-bi(1,3-dithiolylidene)]-4-yl)thio)ethyl (2-chloroethyl)carbamate (8): Compound **8** was synthesized in a similar manner as described in [3, 6]. 2-chloroethyl isocyanate (0.018 g, 0.171 mmol) was added to compound **7** (0.101 g, 0.16 mmol) dissolved in dry and degassed toluene (30 mL). Then the reaction mixture was heated under reflux for 12 h. The solvent was removed in vacuum, and then the residue was dissolved in dichloromethane (50 mL), washed three times with water and dried over anhydrous sodium sulfate. The mixture was concentrated in vacuum and the residue was purified by chromatography on a silica gel column (eluent: petroleum ether/dichloromethane 1:1). Compound **8** was

obtained in 87% yield (0.112 g) as a bright red solid. m.p. 87-89 ^{o}C; ^{1}H NMR [400 MHz, $CDCl_3$, 25 ^{o}C] δ ppm: 4.26 (t, J = 7.0 Hz, 2H, -CH_2OCO-), 3.61 (t, J = 7.0 Hz, 2H, -NHCH_2-), 3.53 (t, J = 7.0 Hz, 2H, ClCH_2-), 3.04 (t, J = 7.0 Hz, 2H, -SCH_2-), 2.81 (t, J = 8.0 Hz, 4H,-SCH_2-), 2.44 (s, 3H, -CH_3), 1.66-1.27 (m, 24H, alkyl-H), 0.88 (t, J = 6.0 Hz, 6H,-CH_3). ^{13}C NMR (100 MHz, $CDCl_3$, 25 ^{o}C): 166.8 (-C=O), 127.9 (-C=C-), 127.8 (-C=C-), 114.1(-C=C-), 106.1(-C=C-), 67.0 (-CH_2O-), 46.4 (ClCH_2-), 40.5 (-NHCH_2-), 31.8 (alkyl-C), 29.2 (alkyl-C), 29.1 (alkyl-C), 28.6 (alkyl-C), 22.7 (alkyl-C), 14.1 (alkyl-C). MS (EI, m/z) = 719.1 (M^+)

General procedure for synthesis of T_1: Compound **T_1** was obtained in a similar manner as described in [3]. A mixture of compound **8** (0.100 g, 0.139 mmol), 4-(methoxycarbonyl)phenol (0.231 g, 1.39 mmol) and potassium carbonate (0.453 g, 2.7 mmol) was dissolved in dry and degassed DMF (30 mL). The reaction mixture was heated at 60 °C for 36 h. The solvent was removed in vacuum, and then the residue was dissolved in dichloromethane (50 mL), washed three times with water and dried over anhydrous sodium sulfate. The mixture was concentrated in vacuum and the residue was purified by chromatography on a silica gel column (eluent: petroleum ether/ethyl acetate 3:1). Compound **T_1** was obtained in 72% yield (0.084 g) as a bright red solid. m.p. 90-91 ^{o}C; FT-IR (KBr, cm^{-1}): v = 3353 (-NH-), 2921 (-CH_2-), 1691 (-C=O); ^{1}H NMR [400 MHz, $CDCl_3$, 25 ^{o}C] δ ppm: 7.99 (m, 2H, phenyl-H), 6.91 (m, 2H, phenyl-H), 5.22 (s, 1H, -NH-), 4.29 (t, J = 8.0 Hz, 2H, -CH_2OCO-), 4.11 (t, J = 6.0 Hz, 2H, -OCH_2-), 3.89 (s, 3H, -OCH_3), 3.63 (t, J = 8.0 Hz, 2H, -NHCH_2-), 3.03 (t, J = 8.0 Hz, 2H, -SCH_2-), 2.81 (t, J = 8.0 Hz, 4H, -SCH_2-), 2.41 (s, 3H, -CH_3), 1.61-1.25 (m, 24H, alkyl-H), 0.88 (t, J = 6.0 Hz,

6H,-CH_3); ^{13}C NMR (100 MHz, $CDCl_3$, 25 ^oC): 166.8 (-C=O), 162.2 (phenyl-C), 155.9 (-C=O), 131.7 (phenyl-C), 123.1(-C=C-), 114.1(-C=C-), 67.0 (-CH_2O-), 53.4 (-OCH_2-), 51.9 (CH_3O-), 40.5 (-NHCH_2-), 31.8 (alkyl-C), 29.2 (alkyl-C), 29.1 (alkyl-C), 28.6 (alkyl-C), 22.7 (alkyl-C), 14.1 (alkyl-C); TOF-MS (ESI, m/z): $[M + Na]^+$ calculated for $C_{36}H_{53}NO_5NaS_8$, 858.1587; found: 858.1590; elemental analysis calculated (%) for $C_{36}H_{53}NO_5S_8$: C 51.70, H 6.39, N 1.67, S 30.67; found: C 51.53, H 6.18, N 1.55, S 30.79.

4,5-Bis((2-hydroxyethyl)thio)-1,3-dithiole-2-thione (9): Compound **9** was synthesized in a similar manner as described in [3]. A mixture of compound **1** (6.015 g, 8.36 mmol) and bromoethanol (3.6 mL, 50.2 mmol) was dissolved in acetonitrile (120 mL). The resulting bright red solution was heated under reflux for 8 h. After cooling to room temperature, the mixture was filtered and the solid was washed with dichloromethane. The combined organic phase was concentrated and the residue was purified by column chromatography (petroleum ether/ethyl acetate 2:1) to give compound **9** as a yellow solid (1.816 g, 75%). m.p. 66-68 ^oC; ^{1}H NMR [400 MHz, DMSO-d_6, 25 ^oC] δ ppm: 3.84 (t, J = 7.0 Hz, 4H, -CH_2OH), 2.96 (t, J = 7.0 Hz, 4H,-SCH_2-). ^{13}C NMR (100 MHz, DMSO-d_6, 25 ^oC): 211.9 (-C=S), 137.8 (-C=C-), 65.1 (-C-OH), 29.5 (alkyl-C). MS (EI, m/z) = 286.1 (M^+).

((2-Thioxo-1,3-dithiole-4,5-diyl)bis(sulfanediyl))bis(ethane-2,1-diyl)bis((2-chloro-ethyl)carbamate) (10): Compound **10** was obtained in a similar manner as described in [3,7]. 2-chloroethyl isocyanate (0.241 g, 2.2 mmol) was added to compound **9** (0.292 g, 1.0 mmol) dissolved in dry and degassed tetrahydrofuran (30 mL). Then the reaction mixture was heated under reflux for 12 h. The solvent was removed in vacuum, and then the residue was

dissolved in dichloromethane (50 mL), washed three times with water and dried over anhydrous sodium sulfate. The mixture was concentrated in vacuum and the residue was purified by chromatography on a silica gel column (petroleum ether/ethyl acetate 1:2). Compound **10** was obtained in 89% yield (0.441 g) as a yellow solid. m.p. 69-71 ^{o}C; ^{1}H NMR [400 MHz, DMSO-d_6, 25 ^{o}C] δ ppm: 4.17 (t, J = 7.0 Hz, 4H, -CH_2OCO), 3.59 (t, J = 8.0 Hz, 4H,-NHCH_2-), 3.34 (t, J = 8.0 Hz, 4H, -CH_2Cl), 3.19 (t, J = 7.0 Hz, 4H, -SCH_2-). ^{13}C NMR (100 MHz, DMSO-d_6, 25 ^{o}C): 211.9 (-C=S), 166.8 (-C=O), 137.8 (-C=C-), 67.0 (-CH_2O-), 46.4 (ClCH_2-), 40.5 (-NHCH_2-), 29.5 (alkyl-C). MS (EI, m/z) = 495.9 (M^+).

((2-Oxo-1,3-dithiole-4,5-diyl)bis(sulfanediyl))bis(ethane-2,1-diyl)-bis((2-chloroethyl)carbamate) (11): Compound **11** was obtained in a similar manner as described in [4]. Mercury acetate (3.501 g, 11 mmol) was added to a solution of compound **10** (1.992 g, 4 mmol) in chloroform/acetate acid (3:1, 60 mL) at room temperature and the mixture was stirred overnight. The mixture was filtered and the solid was washed with dichloromethane. The combined organic phase was washed with water (2 × 30 mL), saturated sodium bicarbonate solution (3 × 30 mL), and brine (50 mL), and then dried over anhydrous sodium sulfate. The organic solvent was removed with a rotavapor to give compound **11** as a faint yellow solid (1.481 g, 77%). m.p. 82-85 ^{o}C; ^{1}H NMR [400 MHz, DMSO-d_6, 25 ^{o}C] δ ppm: 4.14 (t, J = 7.0 Hz, 4H, -CH_2OCO), 3.58 (t, J = 8.0 Hz, 4H,-NHCH_2-), 3.29 (t, J = 8.0 Hz, 4H, -CH_2Cl), 3.16 (t, J = 7.0 Hz, 4H, -SCH_2-). ^{13}C NMR (100 MHz, DMSO-d_6, 25 ^{o}C): 189.9 (-C=O), 166.8 (-C=O), 137.8 (-C=C-), 67.0 (-CH_2O-), 46.4 (ClCH_2-), 40.5 (-NHCH_2-), 29.5 (alkyl-C). MS (EI, m/z) = 479.9 (M^+).

((4',5'-Bis(octylthio)-[2,2'-bi(1,3-dithiolylidene)]-4,5-diyl)bis(sulfanediyl))-bis-(ethane-2,1-diyl)bis((2-chloroethyl)carbamate) (12): Compound **12** was synthesized in a similar manner as described in [6]. The misture of compound **2** (0.410 g, 1 mmol) and compound **11** (0.326 g, 1 mmol) was dissolved in triethyl phosphite (10 mL) and then the mixture was heated at 110 ^oC under nitrogen for 12 h. After cooling to room temperature, the solvent was removed with a rotavapor and the resulting residual was purified by column chromatography (eluent: dichloromethane) to give compound **12** as a bright red solid (0.340 g, 40%). m.p. 85-88 ^oC; ^1H NMR [400 MHz, $CDCl_3$, 25 ^oC] δ ppm: 4.28 (t, J = 7.0 Hz, 4H, -CH_2OCO-), 3.61 (t, J = 7.0 Hz, 4H, -$NHCH_2$-), 3.52 (t, J = 7.0 Hz, 4H, -CH_2Cl), 3.08 (t, J = 7.0 Hz, 4H, -SCH_2-), 2.83 (t, J = 7.0 Hz, 4H, -SCH_2-), 158-1.79 (m, 8H, alkyl-H), 1.43-1.25 (m, 16H, alkyl-H), 0.89 (t, J = 6.0 Hz, 6H, -CH_3). ^{13}C NMR (100 MHz, $CDCl_3$, 25 ^oC): 166.8 (-C=O), 127.9 (-C=C-), 127.8 (-C=C-), 114.1(-C=C-), 106.1(-C=C-), 67.0 (-CH_2O-), 46.4 ($ClCH_2$-), 40.5 (-$NHCH_2$-), 31.8 (alkyl-C), 29.2 (alkyl-C), 29.1 (alkyl-C), 28.6 (alkyl-C), 22.7 (alkyl-C), 14.1 (alkyl-C). MS (EI, m/z) = 854.1 (M^+).

General procedure for synthesis of T_2: Compound $\mathbf{T_2}$ was obtained in a similar manner as described in [3]. A mixture of compound **12** (0.100 g, 0.12 mmol), 4-(methoxycarbonyl)phenol (0.400 g, 2.38 mmol) and potassium carbonate (0.810 g, 5.11 mmol) was dissolved in dry and degassed DMF (30 mL). The reaction mixture was heated at 60 °C for 36 h. The solvent was removed in vacuum, and then the residue was dissolved in dichloromethane (50 mL), washed three times with water and dried over anhydrous sodium sulfate. The mixture was concentrated in vacuum and the residue was purified

by chromatography on a silica gel column (eluent: petroleum ether/ethyl acetate 1:1). Compound $\mathbf{T_2}$ was obtained in 87% yield (0.110 g) as a bright red solid. m.p. 95-96 ^{o}C; FT-IR (KBr, cm^{-1}): v = 3338 (-NH-), 2919 (-CH_2-), 1692 (-C=O); ^{1}H NMR [400 MHz, $CDCl_3$, 25 ^{o}C] δ ppm: 7.99 (m, 4H, phenyl-H), 6.91 (m, 4H, phenyl-H), 4.26 (t, J = 8.0 Hz, 4H, -CH_2OCO-), 4.07 (t, J = 6.0 Hz, 4H, -OCH_2-), 3.89 (s, 6H, -OCH_3), 3.60 (t, J = 8.0 Hz, 4H, -NHCH_2-), 3.08 (t, J = 8.0 Hz, 4H, -SCH_2-), 2.97 (t, J = 8.0 Hz, 4H, -SCH_2-),1.61-1.25 (m, 24H, alkyl-H), 0.88 (t, J = 6.0 Hz, 6H,-CH_3); ^{13}C NMR (100 MHz, $CDCl_3$, 25 ^{o}C): 166.8 (-C=O), 162.2 (phenyl-C), 156.3 (-C=O), 131.7 (phenyl-C), 123.2 (-C=C-), 114.1 (-C=C-), 67.0 (-CH_2O-), 53.5 (-OCH_2-), 51.9 (CH_3O-) 40.2 (-NHCH_2-), 31.8 (alkyl-C), 29.1 (alkyl-C), 29.1 (alkyl-C), 28.6 (alkyl-C), 22.7 (alkyl-C), 14.1 (alkyl-C); TOF-MS (ESI, m/z): $[M + Na]^{+}$ calculated for $C_{48}H_{66}N_2O_{10}NaS_8$, 1109. 2381; Found: 1109. 2378; elemental analysis calculated (%) for $C_{48}H_{66}N_2O_{10}S_8$: C 53.01, H 6.12, N 2.58, S 23.59; found: C 52.83, H 5.98, N 2.35, S 23.63.

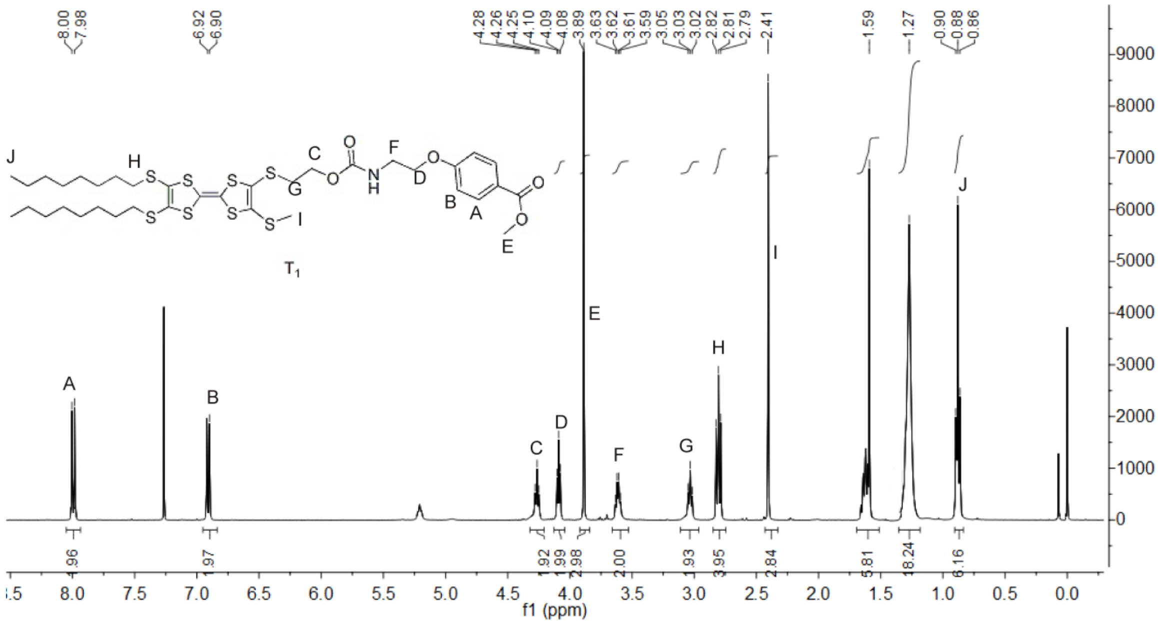

Figure S1: The ^{1}H NMR spectra of $\mathbf{T_1}$ in $CDCl_3$

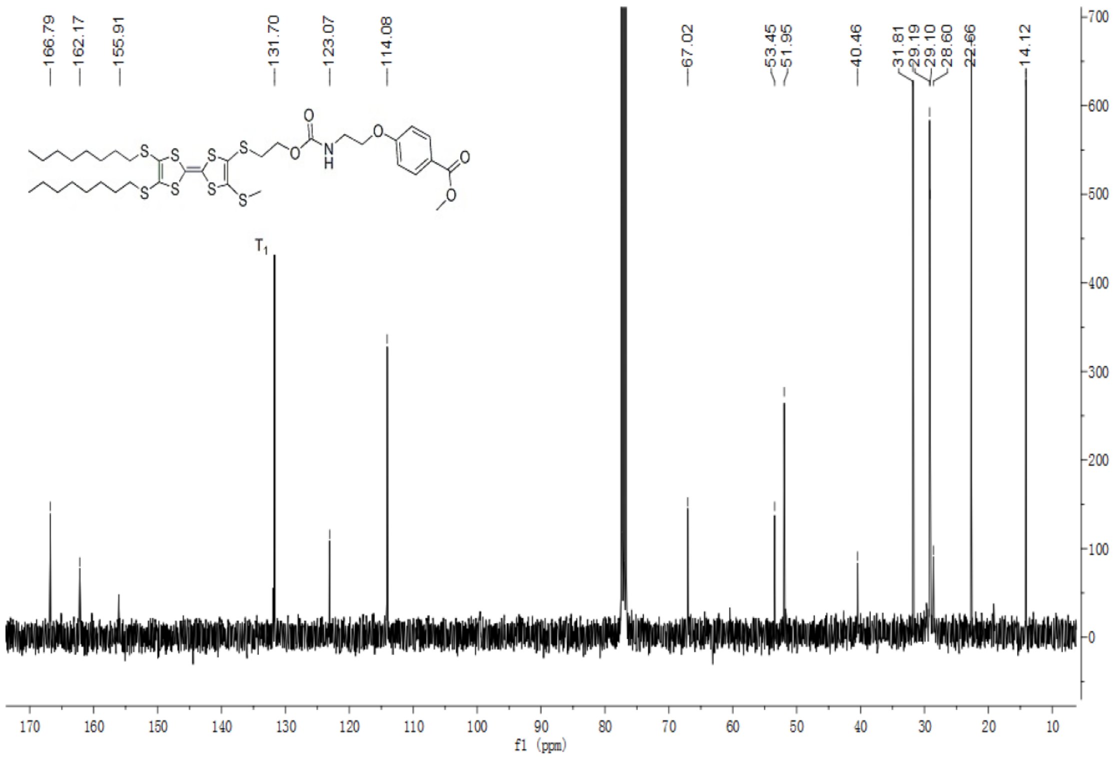

Figure S2: The ^{13}C NMR spectra of $\mathbf{T_1}$ in $CDCl_3$

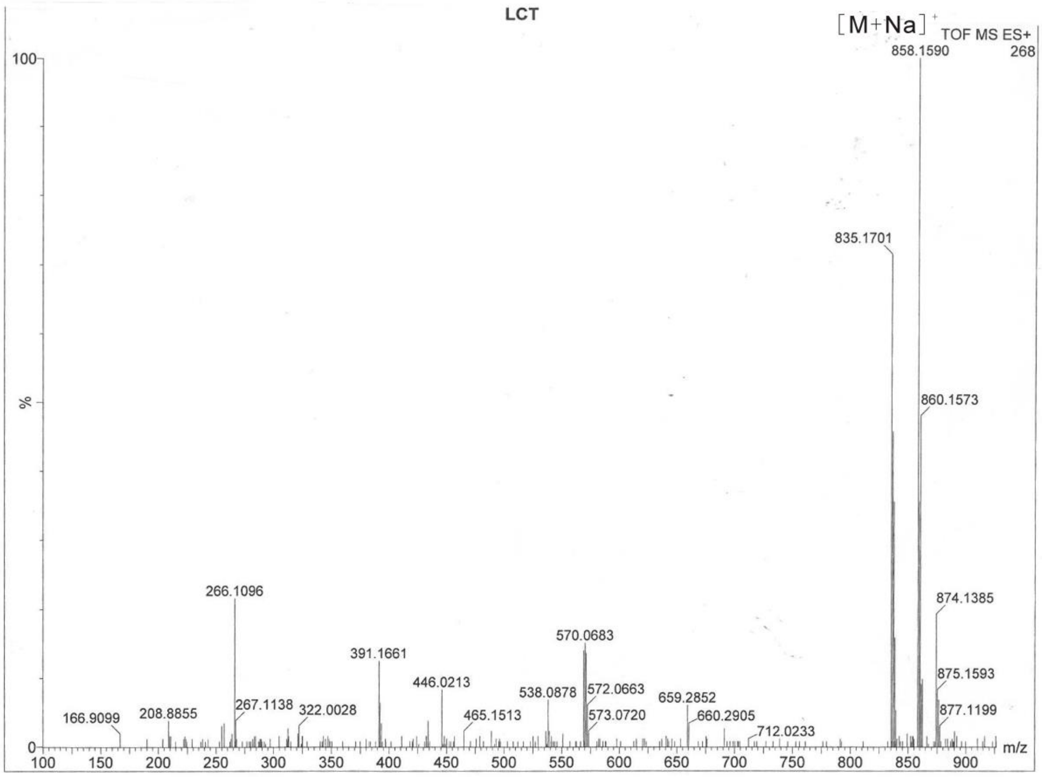

Figure S3: The HRMS of $\mathbf{T_1}$.

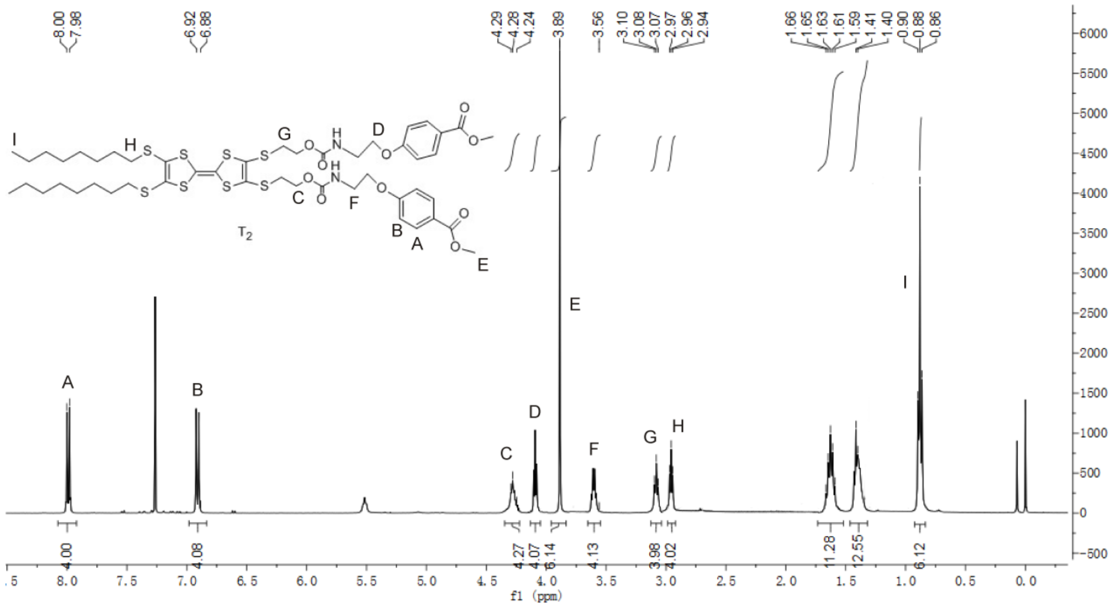

Figure S4: The ^{1}H NMR spectra of $\mathbf{T_2}$ in $CDCl_3$

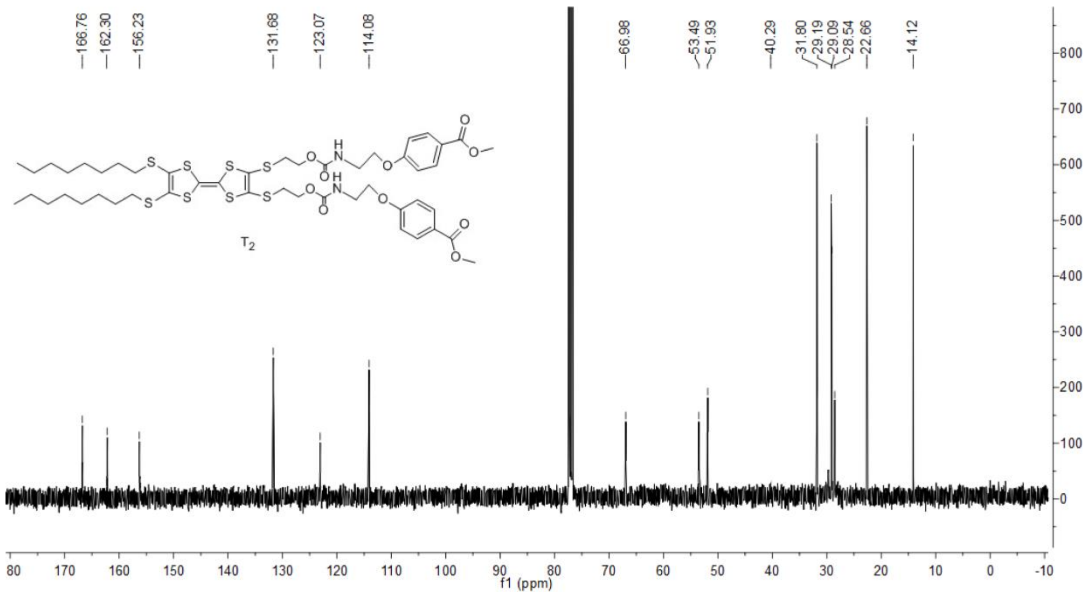

Figure S5: The ^{13}C NMR spectra of $\mathbf{T_2}$ in $CDCl_3$

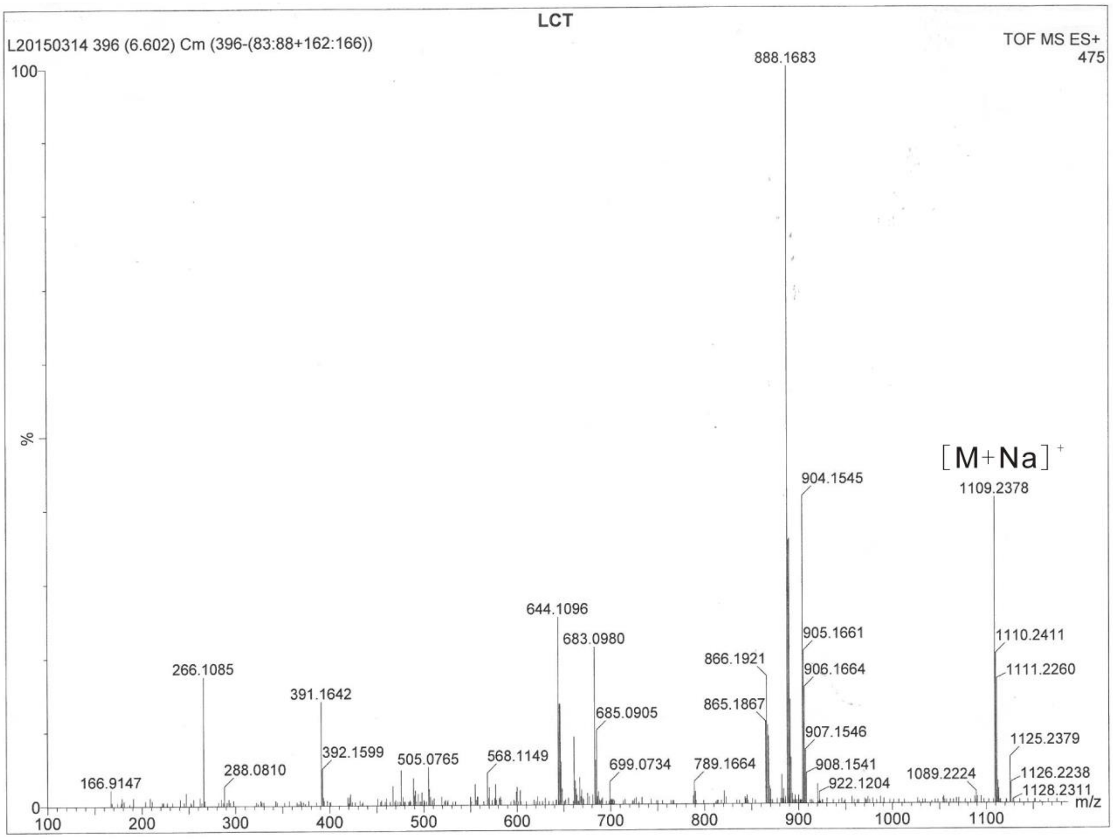

Figure S6: The HRMS of $\mathbf{T_2}$ [8]

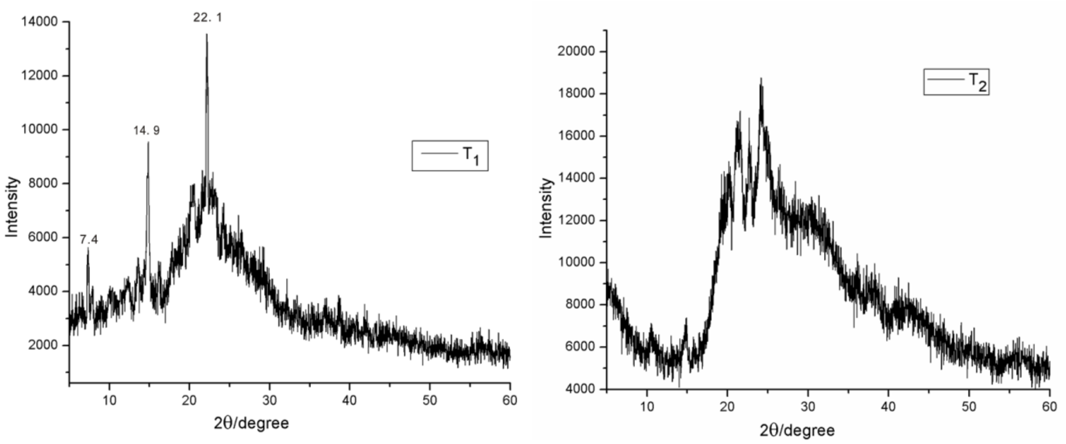

Figure S7: XRD diffraction patterns of $\mathbf{T_1}$ and $\mathbf{T_2}$

Notes and References

[1] Svenstrup, N.; Becher, J. *Synthesis.* **1995,** *3*, 215-235.

[2] Massue, J.; Bellec, N.; Chopin, S.; Levillain, E.; Roisnel, T.; Lorcy, D. *Inorganic Chemistry*, **2005,** *44*, 8740–8748.

[3] Lyskawa, J.; Oçafrain, M.; Sallé, M.; Palacin, S. *Tetrahedron*. **2006,** *62*, 4419-4425.

[4] Zhang, X.; Wang, C.; Lai, G.; Shen, Y. *New Journal of Chemistry.* **2010,** *34*, 318-324.

[5] Benbellat, N.; Gal, Y. L.; Golhen, S. *Synthetic Metals*. **2012,** *162*, 1789-1797.

[6] Tatewaki, Y.; Watanabe, T.; Watanabe, K.; Kikuchi, K.; Okada, S. *Dalton Trans*. **2013,** *42*, 16121-16127.

[7] Adrian, J. M.; Martin, R. B.; Peter, J. S.; *J. Chem. Soc. Perkin trans.* **1993**, *1*, 1403-1410.

[8] The strongest ion in the spectrum is the fragement of $\mathbf{T_2}$ after cracking a urethane group.
